# Development and validation of a nomogram model for lung cancer based on radiomics artificial intelligence score and clinical blood test data

**DOI:** 10.3389/fonc.2023.1132514

**Published:** 2023-03-29

**Authors:** Wenteng Hu, Xu Zhang, Ali Saber, Qianqian Cai, Min Wei, Mingyuan Wang, Zijian Da, Biao Han, Wenbo Meng, Xun Li

**Affiliations:** ^1^ The First Clinical Medical School of Lanzhou University, Lanzhou, Gansu, China; ^2^ Department of Thoracic Surgery, The First Hospital of Lanzhou University, Lanzhou, Gansu, China; ^3^ Saber Medical Genetics Laboratory, Almas Medical Complex, Rasht, Iran; ^4^ Department of Emergency, The First Hospital of Lanzhou University, Lanzhou, Gansu, China; ^5^ Department of Ultrasonography, The First Hospital of Lanzhou University, Lanzhou, Gansu, China; ^6^ Department of General Surgery, The First Hospital of Lanzhou University, Lanzhou, Gansu, China

**Keywords:** lung cancer, artificial intelligence, prediction model, pulmonary nodule, machine learning (ML)

## Abstract

**Background:**

Artificial intelligence **(**AI) discrimination models using single radioactive variables in recognition algorithms of lung nodules cannot predict lung cancer accurately. Hence, we developed a clinical model that combines AI with blood test variables to predict lung cancer.

**Methods:**

Between 2018 and 2021, 584 individuals (358 patients with lung cancer and 226 individuals with lung nodules other than cancer as control) were enrolled prospectively. Machine learning algorithms including lasso regression and random forest (RF) were used to select variables from blood test data, Logistic regression analysis was used to reconfirm the features to build the nomogram model. The predictive performance was assessed by performing the receiver operating characteristic (ROC) curve analysis as well as calibration, clinical decision and impact curves. A cohort of 48 patients was used to independently validate the model. The subgroup application was analyzed by pathological diagnosis.

**Findings:**

A total of 584 patients were enrolled (358 lung cancers, 61.30%,226 patients for the control group) to establish the model. The integrated model identified eight potential factors including carcinoembryonic antigen (CEA), AI score, Pro-Gastrin Releasing Peptide (ProGRP), cytokeratin 19 fragment antigen21-1(CYFRA211), squamous cell carcinoma antigen(SCC), indirect bilirubin(IBIL), activated partial thromboplastin time(APTT) and age. The area under the curve (AUC) of the nomogram was 0.907 (95% CI, 0.881-0.929). The decision and clinical impact curves showed good predictive accuracy of the model. An AUC of 0.844 (95% CI, 0.710 - 0.932) was obtained for the external validation group.

**Conclusion:**

The nomogram model integrating AI and clinical data can accurately predict lung cancer, especially for the squamous cell carcinoma subtype.

## 1 Introduction

Lung cancer is the leading cause of cancer mortality worldwide now. But patients often have a long course without atypical symptoms and signs (1). Therefore, early diagnosis is not possible in most cases, and 5-year survival rate is only 16.1% (2). Low-dose computed tomography (LDCT) is the main method for public physical screening. The tumor markers assessment in hospital including carcinoembryonic antigen (CEA) and cytokeratin 19 fragment antigen21-1(CYFRA21-1) can improve the diagnosis rate (3). Artificial intelligence **(**AI) models are a step forward from automated nodule diagnosis, as they typically do not require nodule measurement or data entry.

Available radioactive prediction models include the Mayo model, Veterans Administration (VA) model, Brock University model, and Peking University People’s Hospital model (PKUPH). However, these models mainly focus on the CT performance of pulmonary nodules and currently, but there is not any model integrating routine blood test data, pathological data and the radioactive models combined with pathological data for accurate prediction of lung cancer (4). In this study, we aimed to build an integrated prediction model for pulmonary nodule diagnosis based on clinical laboratory data and the VA model (5). Thus, we developed a nomogram model incorporating pathological-based subgroup analysis as a timely and efficient tool for clinical application (6).

## 2 Methods

### 2.1 Training population and study design

This retrospective study was conducted in the thoracic surgery department of the First Hospital of Lanzhou University in China, following the Declaration of Helsinki, and was approved by the Ethical Committee of the First Hospital of Lanzhou University (reference number: LDYYLL2021-257). Written informed consent was obtained from all patients. The principles of this study are followed with TRIPOD (The Transparent Reporting of a multivariable prediction model for Individual Prognosis Or Diagnosis).

### 2.2 Patients enrollment

Consecutive pulmonary nodule patients who got AI tool assessment before surgery Between January 2018 to December 2021 were included in this study. Tumor pathological subtypes were assessed by an experienced pathologist. Exclusion criteria were: having accurate pathological data, multiple metastatic tumors, cases with missing data, lung transplant or previous history of lung surgery, and having radiotherapy and chemotherapy.

**Figure 1:**
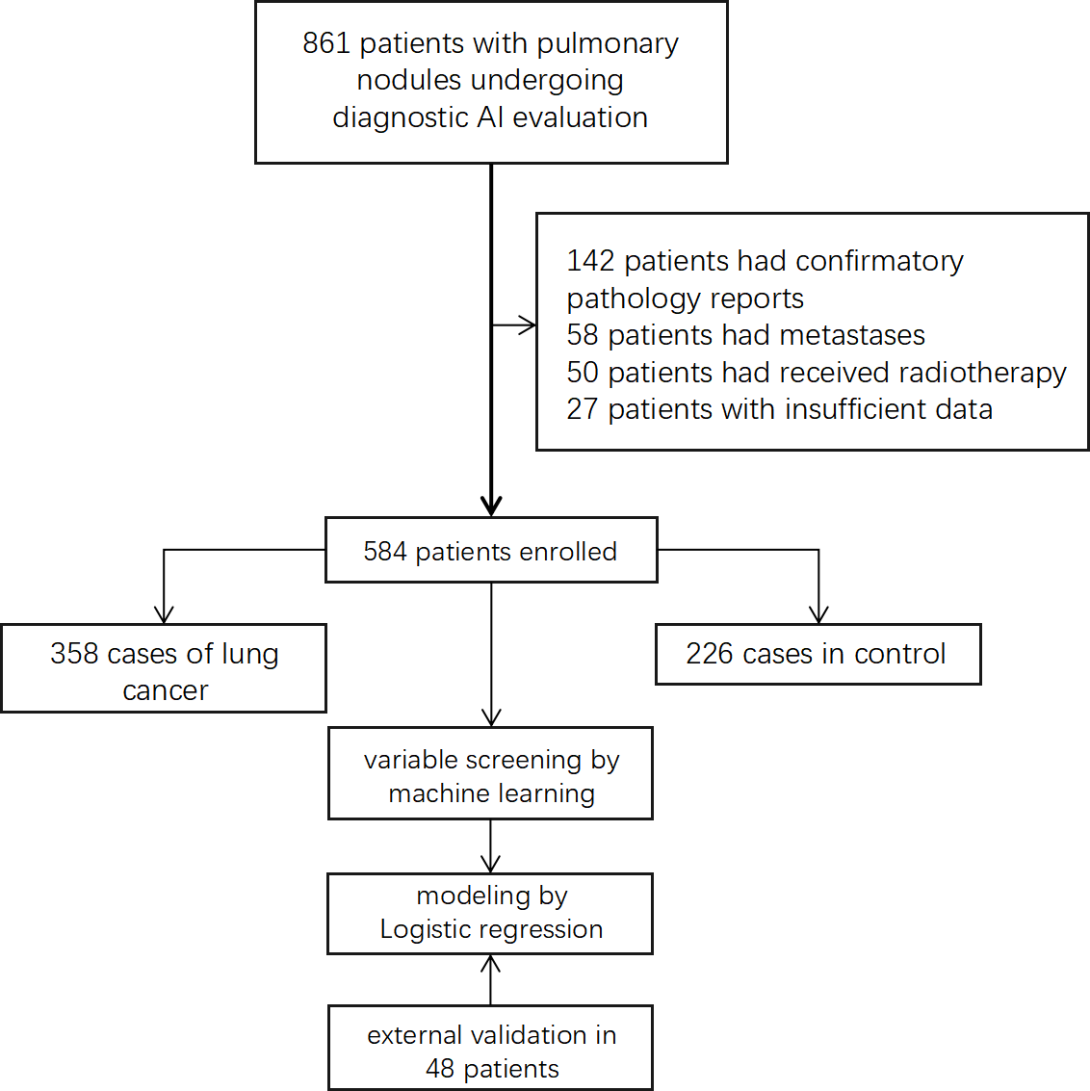
Flowchart of selected patients for modeling.

A total of 861 eligible patients were screened initially. Among these, 142 patients with current clear pathological diagnosis before the operation, 58 multiple metastatic tumors, 27 cases with missing data, and 50 patients with a history of lung surgery, radiotherapy, or chemotherapy were excluded from further analysis. Finally, 584 eligible patients were included in the study to train the model. **Figure 1** shows the flowchart for patient recruitment in this study. In addition, a total of 48 eligible patients from January 2022 to May 2022 were recruited to validate the predictive value of the model.

### 2.3 Data collection

Data was collected independently by two reviewers (WT. H. and X. Z.). More than 76 pre-surgical parameters were evaluated in the developing model: 1. Patients’ basic line characters include age, gender, history of hypertension, diabetes, history of smoking, drinks, and family history. 2. Blood laboratory tests including all parameters of coagulation and blood routine examination data. 3. All routine blood biochemical function test parameters. 4. The lung cancer-associated serum tumor markers include CEA, CYFRA 21-1, squamous cell carcinoma antigen(SCC), neuron-specific enolase (NSE), and Ferritin(FER). 5. The AI assessment score from σ-Discover/Lung Nodule intelligent diagnosis system (the system has got permission from the Chinese Medical Association) with a sensitivity of 80.17% and a specificity of 70.35%. 6. The pathological data include lung cancer subtype, the degree of tumor differentiation, tumor infiltration, the tumor node metastasis (TNM) stage (7).

## 3 Statistical analysis and development of a nomogram

### 3.1 Prediction model development

First, potential risk factors were identified through machine learning methods from the routine blood test data. The selected variables, AI score and patients’ basic line character were used as candidate parameters for model development. Then, the risk variables selection was calculated by stepwise multivariate logistic regression (backward, p<0.05) (8). In addition, different histopathological subtype were analyzed to verify the accuracy of the model for the recognition of different lung cancer subtypes. For clinical application, a nomogram figure was established as an integrated clinical prediction tool.

### 3.2 Machine learning model for variable selection

The machine learning methods were implemented through R (version 4.1.1). The machine learning methods including Least absolute shrinkage and selection operator (LASSO) regression and random forest (RF) were used to identify important features. Lasso regression can handle the multicollinearity problem of the available features and RF enables the implementation of variable selection procedures based on their impact on outcome prediction. RF parameters were optimized in logarithmic steps around their default values (using 500 trees, and a random subspace with dimensionality equal to the rounded value of the square of the number of features). Ten-fold cross-validation and external test set validation were both employed to validate the reliability of the model (9).

### 3.3 Logistic regression model method

Data were analyzed using SPSS v.22.0 (IBM, Armonk, New York, USA). Patients were grouped by postoperative pathological diagnosis. Univariate and multivariate logistic regressions classified the risk factors for lung cancer. The regression models either used chi-square test or student’s t-test for patients’ basic features (age, sex, etc.) analysis. P-values below 0.05 were considered statistically significant. Adjusted odds ratios (ORs) and corresponding 95% confidence intervals (95% CIs) were calculated. The Hosmer-Lemeshow test was used to assess the fitness of the model. The accuracy of the model was checked by plotting DCA(decision curve analysis) curve and CIC(clinical impact curve) curves using predicted probabilities against the actual probabilities. The receiver operating characteristic (ROC) and the area under the curve (AUC) were estimated for discrimination (7).

### 3.4 Subgroup analysis

After model construction, further subgroups analysis according to the postoperative pathological diagnosis. For patients’ pathological diagnosis types including squamous cell carcinoma (SQCC), adenocarcinoma (AD), other tumors such as non-small cell lung cancer (NSCLC), and neuroendocrine tumors patients, The predictive ability of different pathological types of cancer was tested using the integrated model, and the difference between the simple AI predictor and the nomogram was also compared. The prediction performance was estimated by De long test for the AUC, decision curve, and clinical impact curve (10).

### 3.5 External validation

An independent external validation from January 2022 to May 2022 in the First Hospital of Lanzhou University was performed by using the nomogram according to the cut-off parameter. The ROC curve, DCA curve, and CIC curve analysis were performed to validate the accuracy of the model by estimating the difference between the integrated nomogram from the modeling cohort and validation set.

### 36 Role of funding source

The funders had no involvement in study design, data collection, data analysis, interpretation of findings, the writing of this paper, or the decision to submit the paper for publication. There was no commercial support. The corresponding author (WBM) had full access to all the data in the study and had final responsibility for the decision to submit it for publication.

## 4 Results

### 4.1 Patient characteristic

A total of 584 eligible patients were enrolled (**Figure 1**). All patients’ basic line characteristics were analyzed before modeling. There was no difference between lung cancer patients and control group in sex, hypertension, diabetes, family history, drinking, and history of chronic pulmonary diseases before surgery. However, patients with age and smoking history before surgery had a higher rate of carcinoma (**Table 1**).

**Table 1:** All patients’ baseline clinical features analysis.

	Lung cancer (N = 358)	Non-cancer (N = 226)	χ2/Z	*P*
Age (year)	57.65 ± 9.01	48.82 ± 12.73	24.523	0.000
Sex (male), n (%)	205(57.26%)	121(53.54%)	0.779	0.378
Hypertension	57(15.92%)	23(10.18%)	3.260	0.071
Diabetes	38(10.61%)	15(6.64%)	2.656	0.103
Smoking history	114(31.84%)	37(16.37%)	20.8	0.001
Drink history	84(23.46%)	37(16.37%)	1.950	0.153
Family history*	17(4.75%)	4(1.77%)	3.540	0.060
Chronic diseases	108(30.17%)	53(23.45%)	3.013	0.077
Nodule status				
Multiple	126(35.20%)	72(31.86%)	0.688	0.407
Burr around nodule	148(41.34%)	76(33.63%)	3.458	0.062
Nodular calcification	82(22.91%)	34(15.04%)	5.606	0.108
Pleural traction	42(11.73%)	16(7.08%)	3.352	0.067
Solid nodule	125(34.91%)	62(27.43%)	3.824	0.051

### 4.2 Variables selection

**Figure 2:**
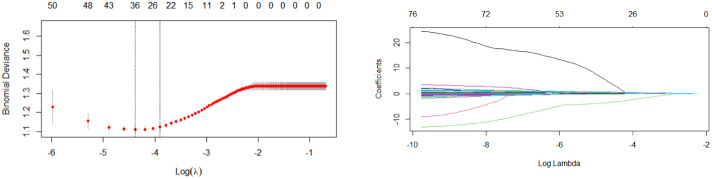
Lasso regression for variable selection.

**Figure 3:**
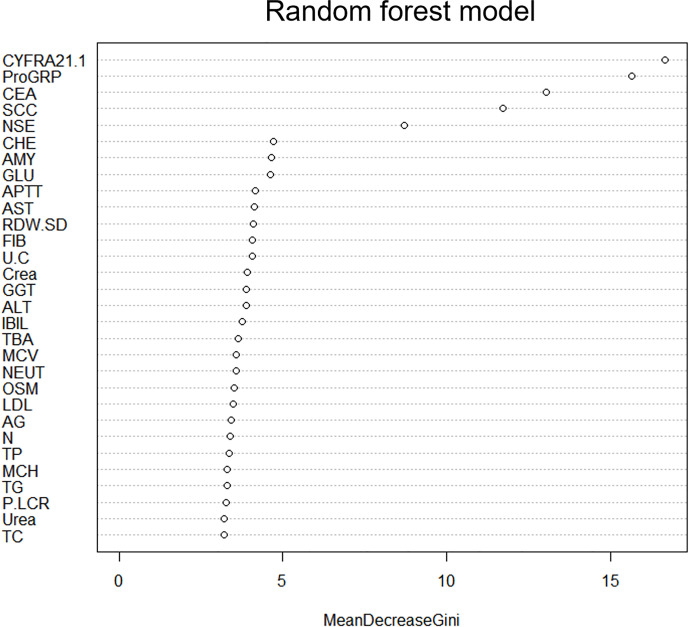
Performance of top-ranking variables selected by RF.

We used the LASSO algorithm to select feature variables from laboratory test data. Except for uric acid (UA), all 76 variables excluded collinearity and could be included in the variable selection using the RF method (**Figure 2**). To obtain the best set of features, the importance of each variable was calculated; 30 features were identified by the RF method finally. These steps were performed by the “RandomForest” package that has been illustrated in **Figure 3**.

### 4.3 Prediction model by logistic analysis

Factors found by the RF algorithms, AI score and baseline data were calculated in the univariate and multivariate logistic regression analysis in training group.

**Figure 4:**
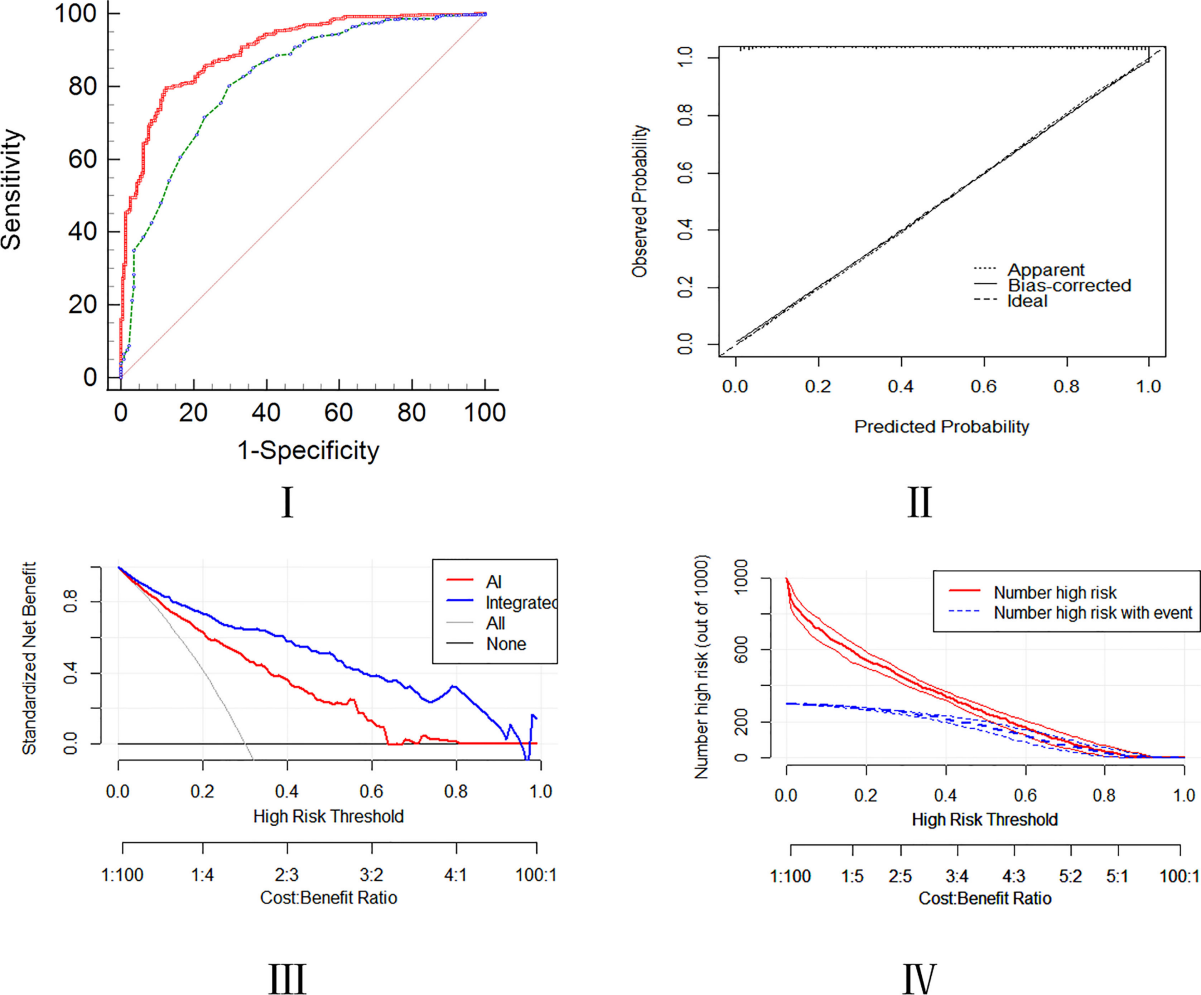
Assessment of predictive ability of the integrated model using the ROC, calibration, DCA, and CIC curves.

For lung cancer patients, radiological AI score higher than 77 (OR=1.098; 95% CI, 1.074-1.123), serum levels of CEA higher than 2.3 µg/L (OR=1.193; 95% CI, 1.019-1.396), serum levels of ProGRP higher than 40.2 µg/L (OR=1.014; 95% CI, 1.001-1.028), serum levels of CYFRA211 higher than 2.5 µg/L (OR=1.714; 95% CI, 1.356-2.167), serum levels of SCC higher than 0.8 U/L (OR=2.336; 95% CI, 1.240_4.402), serum levels of IBIL higher than 16.8 U/L (OR=1.057; 95% CI, 1.009-1.107), the APTT shorter than 34 s (OR=0.916; 95% CI, 0.862-0.974) and age>52 (OR=1.045; 95% CI, 1.018-1.072) were high risk predictors for developing integrated model in lung nodules patients (**Tables 2**, **3**). The AUC of the nomogram for the prediction of lung cancer was 0.907 (95% CI, 0.881-0.929). The De Long test for comparing the performance of the integrated model and the AI model evaluation was 0.001 and accuracy was examined by DCA and CIC curve analysis (**Figure 4**).

**Table 2:** Univariate and multivariate logistic regressions of risk factors for lung cancer.

n/N	Univariate analysis		Multivariate analysis	
	OR (95% CI)	p value	OR (95% CI)	p value
Age (year)	1.080(1.061–1.100)	0.000	1.045(1.018–1.072)	0.001
Smoking history	2.387(1.573–3.620)	0.001		
Hypertension	2.238(1.317-4.115)	0.004		
AI levels	1.099(1.079–1.0119)	0.000	1.098(1.074–1.123)	0.000
CEA	1.516(1.331–1.727)	0.000	1.193(1.019–1.396)	0.028
Cyfra211	1.906(1.607–2.259)	0.001	1.714(1.356–2.167)	0.000
ProGRP	1.033(1.021–1.044)	0.003	1.014(1.001–1.028)	0.038
SCC	4.904(2.882–8.342)	0.000	2.336(1.240–4.402)	0.009
NSE	1.072(1.035–1.110)	0.000		
CHE	0.973(0.883–1.073)	0.588		
AMY	1.008(1.001–1.015)	0.017		
GLU	1.074(0.957–1.204)	0.224		
APTT	0.946(0.909–0.984)	0.006	0.916(0.862–0.974)	0.005
AST	0.978(0.946–0.992)	0.002		
RDWSD	1.045(1.003–1.089)	0.037		
FIB	1.389(1.125–1.715)	0.002		
U/C	0.993(0.986–1.001)	0.081		
Crea	1.026(1.012–1.041)	0.010		
GGT	0.997(0.993–1.001)	0.181		
ALT	0.987(0.978–0.995)	0.003		
IBIL	1.040(1.010–1.071)	0.010	1.057(1.009–1.107)	0.019
TBA	1.001(0.968–1.035)	0.956		
MCV	1.045(1.013–1.078)	0.005		
OSM	1.059(1.021–1.098)	0.002		
LDL	1.384(1.101–1.740)	0.005		
AG	1.038(0.979–1.101)	0.211		
TP	1.000(0.991–1.008)	0.918		
MCH	1.109(1.026–1.199)	0.009		
TG	0.946(0.851–1.051)	0.299		
P/LCR	1.002(0.987–1.016)	0.839		
Urea	1.050(0.935–1.179)	0.041		
TC	1.219(1.031–1.440)	0.020		
N%	1.015(0.997–1.033)	0.103		
NEUT	1.057(0.944–1.184)	0.335		

**Table 3:** The predicted value parameters of risk factors.

Factors	Cut-off	sensitivity	specificity	AUC
Age (year)	52	72.91%	58.41%	0.710
AI levels	77	80.17%	70.35%	0.819
CEA	2.3 µg/L	60.6%	72.1%	0.689
Cyfra211	2.5 µg/L	56.98%	73.34%	0.715
ProGRP	40.2 µg/L	66.76%	64.60%	0.691
SCC	0.8 U/L	55.03%	74.78%	0.703
APTT	34 s	69.0%	42.10%	0.557
IBIL	16.8 U/L	27.6%	94.25%	0.551

### 4.4 External validation of the model

**Figure 5:**
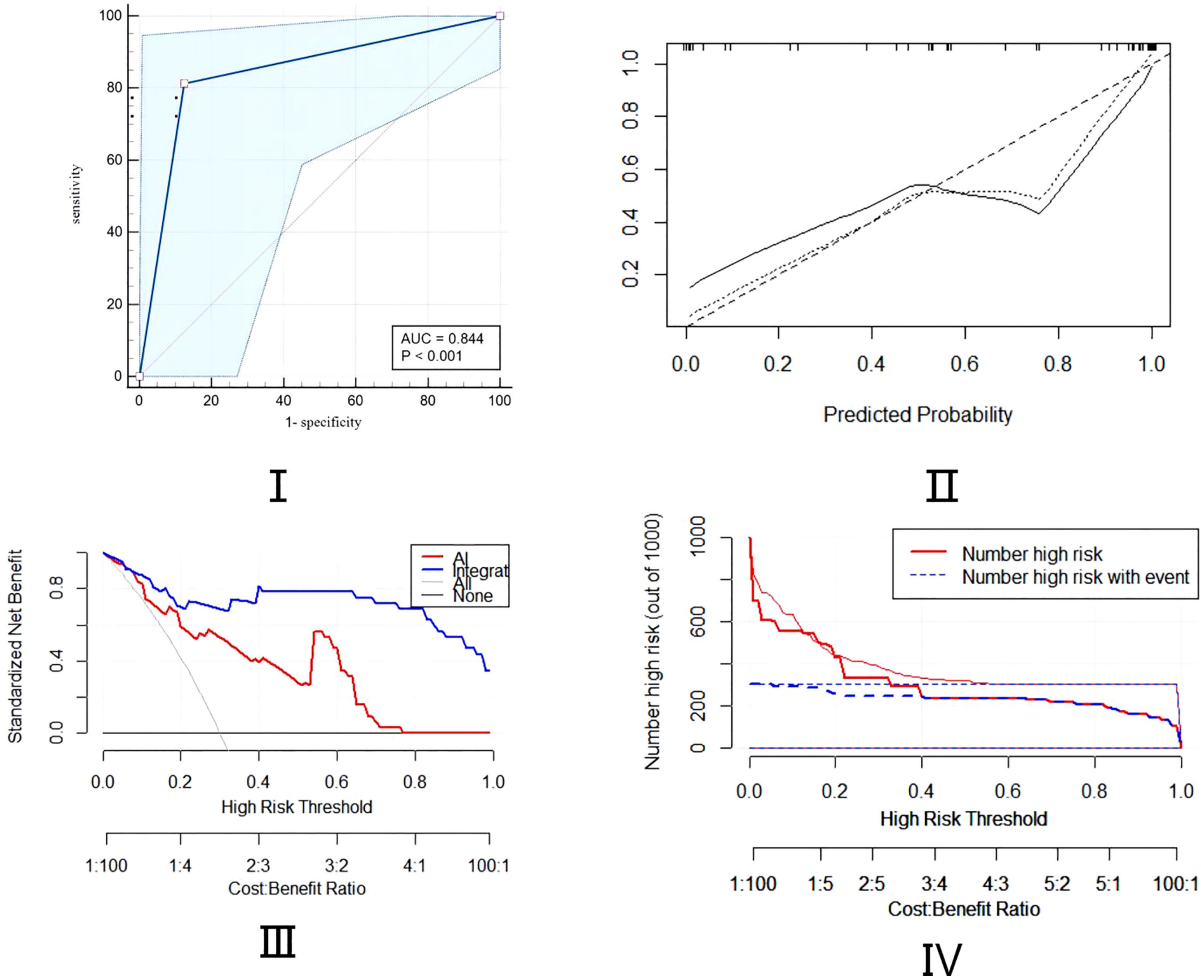
Assessment of predictive ability of the integrated model in external validation cohort using ROC, calibration, DCA, and CIC curves.

A cohort of 48 patients (32 lung cancer,66.7%,16 patients for the control group) was included to validate the nomogram by the cut-off value from the training set, followed by the ROC, DCA, and CIC curve analyses. The prediction ROC curve with an AUC of 0.844 (95% CI, 0.710-0.932)with a sensitivity of 81.20% and a specificity of 87.50%, calibration, DCA and CIC curves showed that the accuracy is in the fitting range. The external cohort showed that our integrated model is in line with the clinical setting (**Figure 5**).

### 4.5 Nomogram of the model

**Figure 6:**
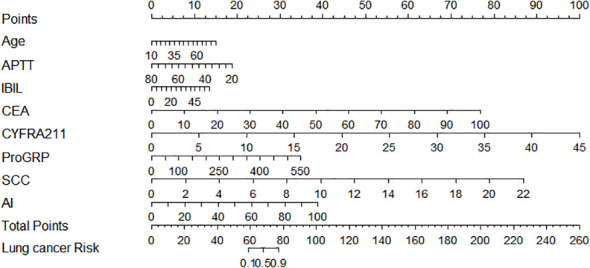
The application of the integrated nomogram model.

To facilitate the application of our model, we established an open access nomogram prediction tool. Users could predict pulmonary nodules by 6 features combined AI scores in the figure (**Figure 6**). Each factor has a prediction reference value based on OR which shows the weight of each parameter and a total score will distinguish between healthy individuals and patients with lung cancer.

### 4.6 Subgroup analysis

The nomogram was analyzed in different histological subtypes of lung cancer. For patients with SQCC, the integrated nomogram model showed a better predictive performance with an AUC of 0.827 (95% CI 0.794-0.857) as compared to the AI model achieved an AUC of 0.668 (95% CI 0.628-0.707). The p-value of the De Long test was 0.001 and accuracy was examined by DCA and CIC curve analysis, which showed the integrated model has a more accurate prediction ability. For patients with AD, the integrated nomogram model showed slightly better predictive performance with an AUC of 0.799 (95% CI 0.764–0.831) as compared to the AI model with an AUC of 0.735 (95% CI 0.697–0.770). The p-value of the De Long test is 0.001 and accuracy was examined by DCA and CIC curve analysis, which showed the integrated model has a more accurate prediction accuracy. For patients with other types of lung tumors, our integrated nomogram model showed no difference in predictive performance with an AUC of 0.728 (95% CI 0.690-0.764) in comparison with the AI model with an AUC of 0.553 (95% CI 0.491-0.574). The p-value of the De Long test was 0.052, the accuracy test in the DCA and CIC curve also proved there is no significant predictive differentiation for the integrated model (**Appendix Figures 1**–**3**).

## 5 Discussion

Lung cancer is the main cause of cancer-related deaths worldwide. Early diagnosis can facilitate intraoperative planning procedures (11). Several risk factors such as age, gender, imaging signs of nodules, and tumor markers are related to the malignancy of pulmonary nodules (12). With the development of artificial intelligence technology, the machine learning models provided a better alternative for creating applicable predictive clinical diagnosis tools. In this study, we developed and validated a diagnostic nomogram model to improve the diagnostic accuracy of lung cancer based on AI tools and clinical data (3, 10, 13).

The integrated model can strongly discriminate between lung malignancies and other pulmonary nodules. The model has the AUC of 0.907, sensitivity of 88.2%, and specificity of 85.3%. In addition, the p-value of the Hosmer Lemeshow test was 0.919, respectively, and the p-value of the De long test with AI was 0.001. The parameters and DCA, CIC, and calibration curve analyses revealed that our integrated model has an excellent predictive accuracy as compared to the AI model only.

The subgroup analysis for different histopathology subtypes demonstrated that for the SQCC, and AD, the integrated nomogram has a more accurate predictive performance advantage compared to the AI. External validation also proved that the integrated model has a better predictive value. We established a convenient and accurate prediction nomogram tool that could be utilized in the clinical setting.

AI systems based on radiomics features, calculated based on the LDCT images, are widely used for the screening and diagnosis of lung cancer. Current studies support the use of AI prediction models as an effective approach for early diagnosis of lung cancer (14). In our study, when the AI assessment score is higher than 77 by the AI system, the risk of lung cancer will increase and the OR has been applied in the nomogram. AI system scoring is the baseline step in this integrated model (15).

Serum tumor markers in serum have great diagnostic value for preoperative diagnosis. CEA, ProGRP, CYFRA211, and SCC can be used in detecting lung cancer; hence, it is necessary to combine serum tumor markers to improve the diagnostic accuracy (6). The integrated model took the application of serum tumor markers more convenient for patients as they are substantially less surgical and cost-effective than other methods. This is consistent with the clinical practice of serum tumor markers angiogenesis and neovascularization in malignancy cancer. Furthermore, the serum tumor markers hold a large proportion in our nomogram, It also suggests that we need to pay more attention to serum tumor markers as prediction parameters for lung cancer (16).

Age is one of the common risk factors for tumor course. In this study, age was positively associated with lung cancer, the optimal cutoff value was 52 years old. Thus, physical examination and screening are necessary for the prevention of lung cancer in the elderly.

Previous studies have shown that indirect bilirubin (IBIL) levels had an influence on survival times in 1,617 patients with resectable lung cancer (17). The optimal cutoff value for serum IBIL was 2.5 µg/L with a sensitivity of 27.6% and specificity of 94.25% suggesting that IBLB inhibits the mTOR pathway by altering the activity of the AMPK pathway leading to lung cancer metastasis. Moreover, APTT is one of the routine indexes of hemostatic examination for surgical patients, the optimal cutoff value for APTT time was 34 S with a sensitivity of 60.6% and a specificity of 72.12% (18). Tumor cell products presented on their surface or substances secreted in the microenvironment may either directly trigger clotting system activation or indirectly trigger it by stimulating extravascular host cells to release procoagulant products (19). This can be also one of the potential targets for cancer detection in the future.

To our knowledge, this is the first diagnostic integrated nomogram model combined with AI tool and clinical blood test data for lung cancer. The validated nomogram showed a high predictive value through the calibration and accuracy test. By the nomogram, the AUC for 8 variables for lung cancer prediction was 0.907 (95% CI, 0.881-0.929), and the p-value of the De Long test is 0.001, which is superior to any single radionics prediction model.

There are still some limitations in our study. First, the nomogram only suits those lung nodule patients instead of routine physical examination for the general population. Secondly, our findings were based on a single-center retrospective study of the eastern Asian population, with an inherent bias with missing data. For future model validation and correction, prospective global multicenter validation and large-scale studies are needed (20).

In conclusion, CEA, AI score, serum ProGRP, CYFRA211, SCC, IBIL, APTT, and age are potential independent factors that can be used for diagnosis of lung cancer. The presented nomogram, as a less invasive and convenient approach, can accurately predict lung cancer in patients with lung nodules, especially for the SQCC subtype to avoid unnecessary surgical resection.

## Data availability statement

The original contributions presented in the study are included in the article/**Supplementary Material**. Further inquiries can be directed to the corresponding author.

## Ethics statement

Written informed consent was obtained from the individual(s) for the publication of any potentially identifiable images or data included in this article.

## Author contributions

The list of full authors in this study is as follows: WH, XZ, AS, QC, MW, MYW, ZD, BH, WM, and XL. Correspondence author: WM. WH, XZ, AS, and WM: protocol development, drafting of this manuscript, critical revision of the manuscript for significant intellectual content. WH, XZ, QC, MW, MYW, ZD, and BH: conducted clinical trials, patient enrollment, and acquired data. WH, BH, XL, and WM: providing personnel, environmental support, and tools and instruments that are vital for the project. WH, XZ, and WM: taking responsibility for statistical analysis, logical interpretation, and presentation of the results. WH, XZ, and WM: taking responsible for pathology and figures. WH, XZ, AS, QC, MW, BH, and WM: Review the article before submission not only for spelling and grammar but also for its intellectual content. WH, XZ, WM, and XL: constructing an idea or hypothesis for the manuscript, providing critical revision. WH, XZ, and WM accessed and were responsible for the raw data and the models in the study. All authors contributed to the article and approved the submitted version.
