# Supplementary figures and images for: Development and validation of a nomogram model for lung cancer based on radiomics artificial intelligence score and clinical blood test data
Source: Front Oncol. 2023 Mar 29;13:1132514. doi: 10.3389/fonc.2023.1132514 (PMC10090418; doi:10.3389/fonc.2023.1132514)

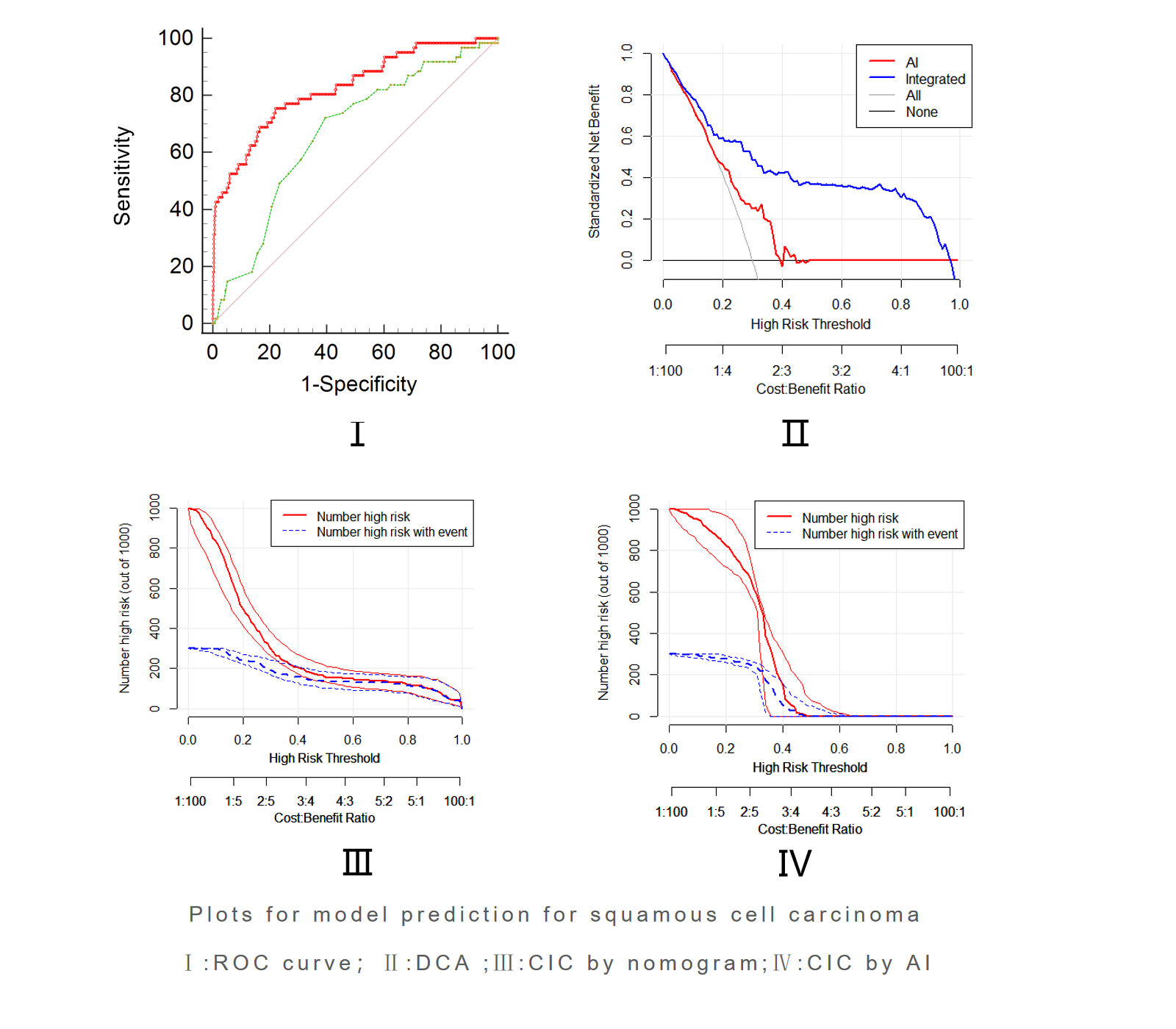

Supplement: Supplementary file 1 [file Image_1.tif]

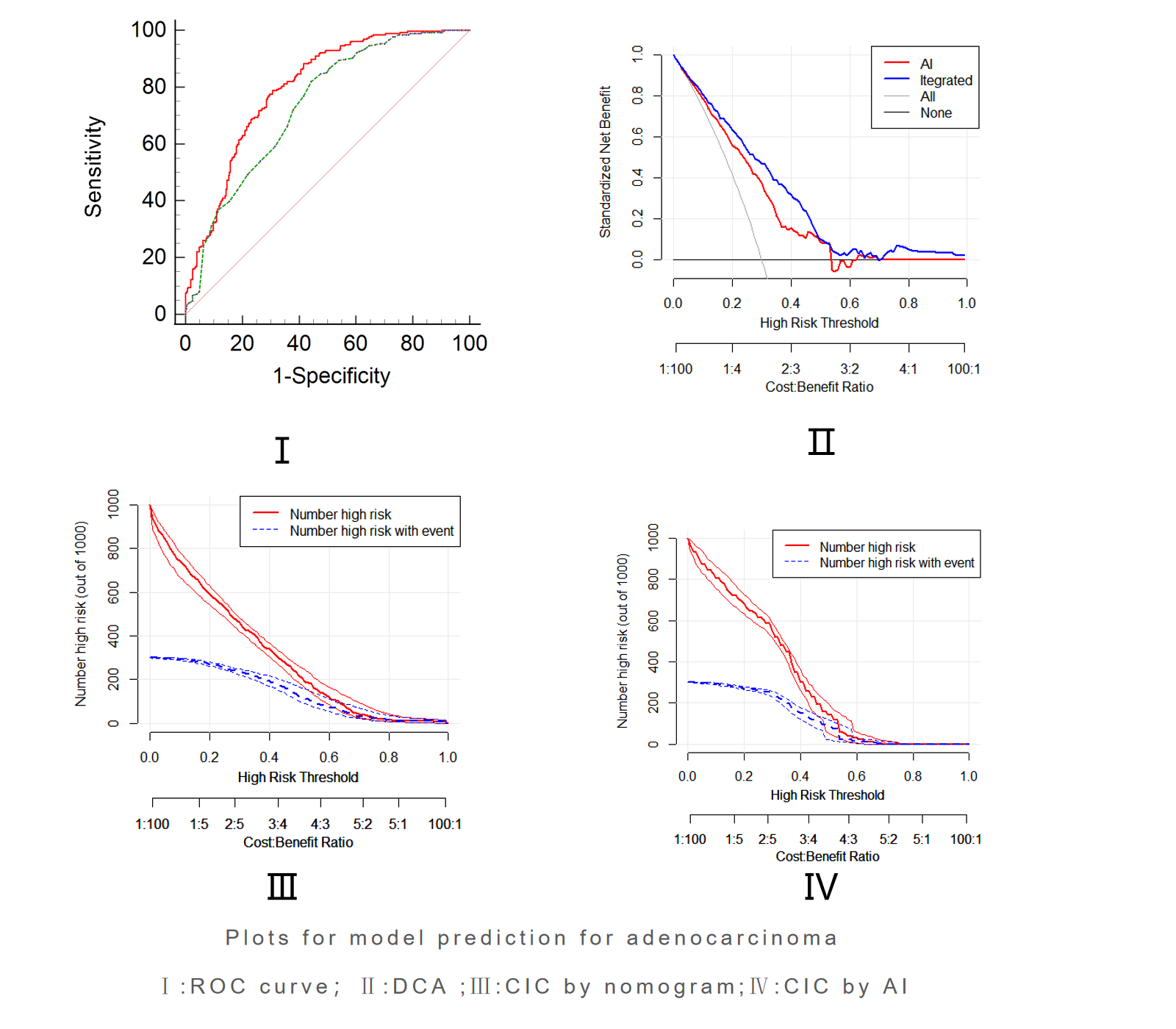

Supplement: Supplementary file 2 [file Image_2.tif]

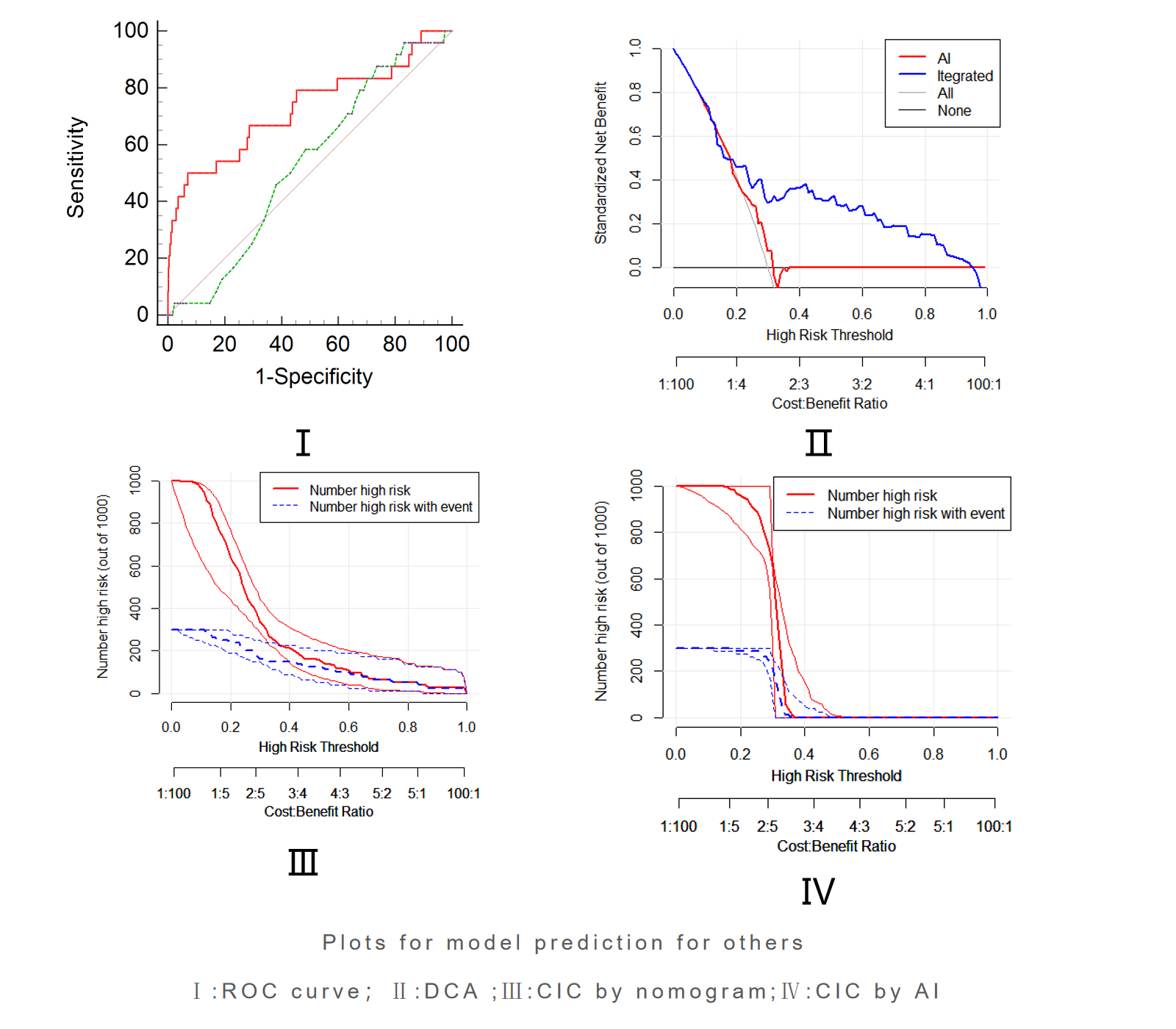

Supplement: Supplementary file 3 [file Image_3.tif]
